# Marshallese Mothers’ and Marshallese Maternal Healthcare Providers’ Perspectives on Contraceptive Use and Reproductive Life Planning Practices and Influences

**DOI:** 10.3390/ijerph20053949

**Published:** 2023-02-23

**Authors:** Britni L. Ayers, Rachel S. Purvis, Jennifer Callaghan-Koru, Sharon Reece, Sheena CarlLee, Nirvana Manning, Krista Langston, Sheldon Riklon, Pearl A. McElfish

**Affiliations:** 1 College of Medicine, University of Arkansas for Medical Sciences Northwest, 2708 S. 48th St., Springdale, AR 72762, USA; 2 College of Medicine, University of Arkansas for Medical Sciences Northwest, 1125 N. College Ave, Fayetteville, AR 72701, USA; 3 College of Medicine, University of Arkansas for Medical Sciences, 4301 W. Markham St, Little Rock, AR 72205, USA; 4 Office of Community Health and Research, University of Arkansas for Medical Sciences Northwest, 2708 S. 48th St, Springdale, AR 72762, USA

**Keywords:** contraception, reproductive life planning, Marshallese, healthcare providers, maternal and child health, Pacific Islanders

## Abstract

Pacific Islander communities experience significant maternal and infant health disparities including high maternal and infant mortality. Contraception and reproductive life planning prevent approximately one-third of pregnancy-related deaths and neonatal deaths. We report the results of formative research devoted to understanding Marshallese mothers’ as well as their maternal healthcare providers’ practices and influences related to contraceptive use and reproductive life planning. This study used an exploratory, descriptive qualitative design to explore Marshallese mothers’ and maternal healthcare providers’ practices and influences of contraception use and reproductive life planning. Twenty participants were enrolled in the study, 15 Marshallese mothers and five Marshallese maternal healthcare providers. For the Marshallese mothers, two themes emerged: (1) Reproductive Life Planning Practices and Information; and (2) Reproductive Life Planning Influences. For the Marshallese maternal healthcare providers, two themes emerged: (1) Reproductive Life Planning Practices; and (2) Reproductive Life Planning Influences. This is the first study to document Marshallese mothers’ and maternal healthcare providers’ practices and influences with contraceptive use and reproductive life planning. Study results will inform the development of a culturally-adapted contraception and reproductive life planning tool with an educational program for Marshallese family units and maternal healthcare providers serving Marshallese women.

## 1. Introduction

Pacific Islander communities experience significant maternal and infant health disparities [1,2,3,4,5,6,7,8]. Pacific Islanders have almost twice the infant mortality per 1000 live births of non-Hispanic whites and have a higher maternal mortality compared to non-Hispanic whites (13.5 verse 12.7) [9]. One of the fastest growing communities of Pacific Islanders in the United States (US) is the Marshallese community in Northwest Arkansas [10,11,12,13,14]. Marshallese in Arkansas also experience a high rate of adverse perinatal outcomes: 19% of Marshallese infants were born preterm (compared to 9.6% nationally), and 15% of Marshallese were low-birth-weight (compared to 8.3% nationally) [8,15,16]. These disparities may be confounded by limited contraception and reproductive life planning. Reproductive life planning is a set of personal goals about having or not having children.

Contraception and reproductive life planning can prevent approximately one-third of pregnancy-related deaths and 44% of neonatal deaths [17]. This is largely due to education surrounding the timing and spacing of pregnancies. It is one of the most effective ways to reduce the risk of adverse maternal and infant health outcomes [18]. Reproductive life planning, according to the World Health Organization, allows individuals and couples to anticipate and attain their desired number of children and the spacing and timing of their births, through the use of modern contraceptive methods [18]. Ensuring access and providing education about sexual and reproductive health services, including contraception and reproductive life planning, can be a cost-effective approach to prevent maternal and infant disparities [19,20].

There is limited data on the use of contraceptives and reproductive life planning within Pacific Islander communities. A systematic review documented factors influencing low contraception use patterns among Torres Strait communities and identified a lack of access to culturally appropriate health services, discomfort with condoms, and reproductive coercion by partners (e.g., partner wants a baby) [21]. Separate studies with Marshallese and Native Hawaiians identified an overall lack of knowledge of contraceptive options, cultural customs of not discussing contraceptive use and reproductive life planning methods, and cultural views that pregnancies are considered a blessing [22,23]. 

To help address maternal and infant health disparities in the Marshallese community, the University of Arkansas for Medical Sciences has implemented the Healthy Start program [24]. The program, which is described elsewhere [24], focuses on ensuring early prenatal care, well woman care (including contraceptives), and interconception health. Recognizing short intervals between pregnancies as a potential contributor to poor birth outcomes among Marshallese community members [24], the study team sought to understand contraceptive use and reproductive life planning among Marshallese in Arkansas. 

## 2. Methods

### 2.1. Research Design

This study used an exploratory, descriptive qualitative design to explore Marshallese mothers’ and maternal health care providers’ (MHCPs) practices and influences of contraception use and reproductive life planning. While the focus of the study was qualitative, a quantitative survey was used to capture demographic data including relationship and pregnancy status. A community-based participatory research (CBPR) approach was followed to design and implement the study. CBPR methods help to ensure cultural appropriateness of research methods through the collaboration between academic partners and the community [25]. For this study, the Healthy Start Community Action Network (CAN), comprised of local Marshallese community members and healthcare professionals, was engaged in all aspects of the study. Several members of the interprofessional research team are Marshallese. All study plans and documents—including recruitment plans and materials, consent documents and forms, retention plans, quantitative surveys that document participant demographic information, and qualitative interview guides—were developed in partnership with the CAN and Marshallese research team members. This study and all procedures involving research study participants were approved on 25 June 2021 by the University of Arkansas for Medical Sciences Institutional Review Board (Protocol #26297).

### 2.2. Participant Eligibility, Consent, and Recruitment

Participants were eligible to participate in the study if they were 18 years of age or older and met the criteria for one of the following groups: (1) employed as a healthcare professional and caring for Marshallese women or those who self-identified as Marshallese, or (2) females of reproductive age (under 49 years of age) who self-identified as Marshallese and were pregnant in the past 12 months. Marshallese bilingual female study staff recruited participants during women’s health appointments, maternal health home visits, and community events. Potential participants who met the inclusion criteria were offered the opportunity to join the study and complete the consent process. Trained study staff read the consent aloud to the potential participant in their language of choice (English or Marshallese) and provided participants with a study information sheet. Participants had the opportunity to ask questions prior to verbally providing consent. Participants were provided with a copy of the information sheet used to obtain verbal consent. Interviews took place in a community setting or the participant’s home. 

### 2.3. Instrumental Development and Data Collection

Data were collected from August to October of 2021. The CAN and bilingual study staff co-developed, reviewed, edited, and approved the demographic survey and qualitative interview guide prior to data collection. The survey was implemented using the web-based Research Electronic Data Capture (REDCap) system (Vanderbilt University, Nashville, TN, USA) [26] and took approximately 15–30 min to complete. The qualitative interview guide went through two iterations and took approximately 30 to 60 min to complete (see Supplementary Material S1). All interviews were conducted by trained bilingual Marshallese study staff. Interviews were audio recorded and then transcribed in the language spoken by participants. Transcripts in Marshallese were then translated into English by professional translators and checked for accuracy by Marshallese-speaking study staff. A total of 20 participants—15 Marshallese mothers and five Marshallese MHCPs—were enrolled in the study. Marshallese mothers that completed the survey and interview received a $40 gift card. 

### 2.4. Data Analysis

Demographic information was summarized, and descriptive statistics are presented with frequencies and percentages. Interview transcripts were thematically analyzed following an inductive approach. Three qualitative researchers (two main coders, one confirmation coder) began with initial coding, which consisted of naming each data segment with short summations. The open codes were elaborated and refined into a final codebook through consensus discussions among the coders. All transcripts were coded according to the final codebook, and the frequency and patterns of codes were reviewed to identify and develop the most salient categories within the data [27]. The research team discussed the emergent themes to ensure scientific rigor and inter-coder agreement. Marshallese are a collectivist culture (value group over individual) and tend to use words like “us” rather than “I.” This is reflective in some of their quotes [28,29].

## 3. Results

A total of 15 Marshallese mothers (Table 1) and five Marshallese MHCPs (Table 2) were enrolled in the study. Results of the mothers and MHCPs are presented separately.

### 3.1. Marshallese Mothers Demographic Characteristics

Table 1 shows the Marshallese mothers’ demographic characteristics. All of the mothers were Marshallese and not pregnant. A majority of the mothers were in an unmarried partnership (66.7%). The mothers’ ages ranged from 20 years old to 42 years old, and the mean age was 30 years old with a standard deviation of 7. Most of the mothers (80%) had completed high school and some college or technical school.

### 3.2. Marshallese Mothers Qualitative Results

For the mothers, two themes emerged: (1) Reproductive Life Planning Practices and Information; and (2) Reproductive Life Planning Influences. Illustrative quotes and participant identification (PID) numbers are presented below.

#### 3.2.1. Reproductive Life Planning Practices and Information

The mothers discussed their contraception and reproductive life planning practices and where they received their information. 

Reproductive Life Planning Practices. When the mothers were asked about contraception and reproductive life planning practices, some mothers reported using no contraception and/or reproductive life planning method. Some mothers stated, “I’ve never done something like this;” (PID 2) and “Oh, I’ve never done this” (PID 22). Others reported knowledge of contraception and reproductive life planning but had not utilized it for themselves: “They know there is birth control but they don’t use it,” (PID 5) and “For most of us Marshallese, some of us don’t use controls, so our kids end up being probably a year or not even a year apart” (PID 17). One mother stated, “I think in our Marshallese community, we don’t talk about it. I feel like in our culture, they were not open to talking to each other about reproductive life planning” (PID 01).

The mothers reported they felt others in the Marshallese community might choose contraception if they did not want children at that time. One mother said: “Yes, probably the ones that probably don’t want kids yet. I would think they would probably be open with their options on what to take and what could last longer for them” (PID 17). Another mother echoed this sentiment, and this was predicated on the workload of having additional children. She said: “If they were wanting to space out the years between their kids, so that it can also help them and make it easier, because kids are a handful” (PID 17).

While participants describe a lack of contraception and reproductive life planning information and lack of contraceptive use in the community in general, when asked about their own understanding and their family and friends’ understanding of contraceptives, several participants described knowledge of a wide range of contraceptive methods. Participants said: “I would say the tube [Nexplanon]. I have some families, they use the tube” (PID 17). Another mother said: “Something like the tube [Nexplanon], the shot/vaccine, pill, and tube tied” (PID 20). One mother said: “The shot and the tube. The tube in your arm” (PID 25). Another mother said: “Pills, shot, and the withdrawal-type” (PID 3). One mother said: “The shot, pills, using condoms” (PID 26). Only one mother discussed the use of intrauterine devices (IUDs) when she said: “I’ve heard about IUDs a lot. I have a lot of friends that use IUDs and just a couple that use the implants. I don’t know a lot of people that do just the pill” (PID 1). 

Mothers expressed that decisions about contraception and reproductive life planning should be a woman’s choice: “I think it should be up to our self, because it depends on how we want our life to play out, if we want kids, or we want to hold off on that. How you control your body is up to you” (PID 17). Additionally, other mothers stated: “I think you just make that choice of your own. Then you can talk to your healthcare provider with any type of what your options are and what’s best fit for you” (PID 1). Another mother said: “The woman herself. She’s the only one that can prevent it. It’s her choice” (PID 3).

Reproductive Life Planning Information. A majority of the mothers described obtaining information about contraception and reproductive life planning from their MHCPs. Mothers said they got their information from: “Healthcare providers” (PID 2); “The doctors” (PID 24); “I heard from my doctors” (PID 25); “The doctors” (PID 26); and “From the doctor” (PID 3). Mothers described getting this information during their prenatal visits, stating, “During second pregnancy, my doctor explained it to me,” (PID 23) or at their postpartum visit:

*I also heard about IUD from my OB-GYN (obstetrician-gynecologist) when I went for my follow up after giving birth. I learn a lot from my OB-GYN because he gave me a pamphlet of risk factors and all of that and how it works. (PID 01)*

In addition, mothers described learning from online videos or through social media outlets. For example, one mother said: “They learned online. They would watch videos online” (PID 25). Another mother said: “The ones that I learned about, I just come across on Facebook” (PID 17).

Some mothers discussed learning about contraception and reproductive life planning methods from family and friends. One mother said: “Also, friends and family; you talk about what kind of birth control they have and what kind of experience they have with it. It’s how I choose my birth control method” (PID 1). Other mothers said: “My family. My sisters, yes” (PID 20); “I’ve heard IUDs from a lot of friends” (PID 1); and “From my friends, families” (PID 26).

#### 3.2.2. Reproductive Life Planning Influences

The mothers explained influences on contraceptives and reproductive life planning, and five subthemes emerged: (1) Partner/Family; (2) Religion; (3) Side Effects; (4) Cost; and (5) Fear.

Partner/Family. Mothers consistently described their partners as a strong influence in their contraception and reproductive life planning decisions. For example, when asked who influenced contraception and reproductive life planning choices, mothers said: “My husband” (PID 26); “Husbands/partners” (PID 23); and “Partners/husband or family members, like our moms and dads” (PID 02). Another participant stated, “I feel that in our community, it’s more of just between one’s immediate family, between the husband and a wife where there’s vows” (PID 01). Another mother echoed this sentiment: “Their husbands/partners, yes. We have to let them know though or they will wonder why we can’t get pregnant, and we have to let them know before we take birth controls” (PID 20). The women discussed that the support from their partners with regard to contraception and reproductive planning varied. One mother said: “Our husbands. Some would be supportive and some wouldn’t. Their husbands don’t want them on birth control” (PID 24). Another mother said: “He [referring to partner] doesn’t like birth control” (PID 04). Another mother said that “men” (PID 05) were the strongest influence for reproductive life planning beliefs and practices. 

Religion. Mothers also discussed how religion was a dominant influence in contraception and reproductive life planning within their community. Mothers said: “Religions” (PID 02); “Because some [those that are religious], they don’t believe in birth control” (PID 03); and “Their religion could prevent them from taking those birth controls” (PID 25). Another mother said: “I think religious people may be. That’s the only one I can think. I know as Catholic, we’re not supposed to use any type of birth control except for the calendar” (PID 01). One mother specifically said: “Those that go to church and they are faithful” (PID 25). Some of the mothers described the use of contraception as connected to sin and unmarried sex. One mother said: “Christians. I think that some would be against it or some like families or people at church would be against it because they think a girl is planning to sin” (PID 05).

Side Effects. Another dominant influence that emerged in discussing contraception and reproductive life planning was the potential for side effects of contraceptives. One mother said: “Probably, because of the side effects, or because of what could happen to their body out there, getting it implanted in them” (PID 17). Other mothers agreed and said: “They are concerned because they say they have headaches, some would sleep a lot, hair loss, and plenty others reasons” (PID 24). Another mother said: “Because some say they gained weight, for some, they skin becomes darkened, and they just feel sick with it” (PID 25). And one mother said: “I hear a lot from those that use birth control that they get bigger from it, it changes their appetite and stuff” (PID 05).

The discussion around potential side effects was also interwoven with concerns of long-term effects. One mother said:

*People telling the story about getting stuff implanted in them. Then they end up getting their skin damaged or their body damaged, because they have to look for the implant inside of them, because it got lost. I learned about it on Facebook, people sharing their stories about what happened to them when they lose the implant inside of their body. Then, I also have a friend that used to have this same birth control. She had one implanted in her and when she first put it in there, they were able to feel it. A few years later, she told me, she couldn’t feel it anymore, but she never told me like, if she went to get it removed, or if she still has it in there. (PID 17)*

Another mother said: “I feel like whenever I would tell a family member about it, they’re—the first thing they think is, ‘Oh, they’re going to put that in there? Wouldn’t that cause cancer?” (PID 01). Additionally, there was concern of infertility in the future. One mother said, “The only thing I know they’re concerned of is, usually they say when they take any birth control, it’s harder for them to have kids. Yeah, after they take it out” (PID 3). Another mother said: “I have aunts who tell me not to take birth control because they say it messes my system up, and in the future, it’ll be hard for me to conceive” (PID 03).

Cost. Another influence on contraceptive use and reproductive life planning was the cost, as Marshallese non-pregnant women have not qualified for Medicaid/Medicare until December 2020. Marshallese residing in the US are Compact of Free Association (COFA) migrants, meaning Marshallese may migrate from the Marshall Islands to the US but are not considered US citizens. Therefore, Marshallese COFA migrants did not qualify for health insurance prior to this policy change in 2020 [30]. Mothers discussed how the lack of insurance, coupled with lack of money, was a strong influence in accessing contraceptives. One mother said: “The cost of it, depending on what kind of birth control they want. That’ll probably be the only thing preventing them from getting it” (PID 17). Another mother said: “In our community, not having insurance makes it hard to go to an appointment” (PID 2). 

Lack of money and/or insurance was also interwoven with the primary care visits. One mother said: “It could be because they don’t have enough money to pay for the procedure” (PID 26). Another mother said: “They don’t go to the doctor’s as often or some just don’t have insurance” (PID 3).

Fear. Lastly, Marshallese mothers described fear as an influence to accessing contraception, and much of this was rooted in the concept of inserting a foreign object into their bodies. For example, one mother said: “Some are concerned because of the thin rod, and they are afraid when they inserted under their skin” (PID 24). Another mother said: “I would say, something that they put into your body. I think that probably would scare some women” (PID 01). Another mother said: “Some don’t get it because they are scared” (PID 05). The mothers explained their family members discouraged them from using birth control using fear. For example, one mother said: “If they talked to their family members and they tried to make them scared” (PID 25).

Fear of contraception was also embedded in fear of their doctor. One mother said, “They don’t know if the doctor doing the implants is experience enough to do the job, could it be implanted wrong, these kind of things” (PID 20). This fear also extended to fear in discussing the process with their doctor. One mother said, “A lot of Marshallese women will go into their follow up and not even ask questions because they’re too afraid or don’t know what to say” (PID 01). 

### 3.3. Maternal Healthcare Providers’ Demographic Characteristics

Table 2 shows the MHCPs’ demographic characteristics and information about their practice and facilities. A majority of the MHCPs were female, and all of the MHCPs were Marshallese. The MHCPs specialties varied from clinical nurse (2), outreach specialist (1), disease intervention specialist (1), and family medical doctor (1). 

For the MHCPs, two themes emerged: (1) Reproductive Life Planning Practices; and (2) Reproductive Life Planning Influences.

#### 3.3.1. Reproductive Life Planning Practices 

MHCPs discussed their perceptions of Marshallese women’s contraception and reproductive life planning practices and influences. The majority of the MHCPs stated that Marshallese women did not use contraception and reproductive life planning practices. For example, one MHCP said:

*I don’t think they know; I don’t think they use birth control at all … I don’t think they’re using it, or at least from when I’ve asked some of the girls I see. I don’t think I have ever come across anybody that uses birth control, to be honest. That’s just me speaking from work experience. Not from the ones I know of, the ones around me. I feel like, with experiencing the work in the labor and delivery, I feel like it’s never planned. I don’t think they ever talk about having kids, so I think it just happens. (PID 1)*

Contraception and reproductive life planning practices appear to vary generationally. One MHCP stated:

*I think it depends on the generation. The older generation, I think, I don’t know, they might—from what I’ve learned, and what I’ve seen in the islands, they don’t plan it. I mean, they just have children. Yeah. I’ve seen the younger generation not because of more education on family planning, they get together and they talk about it. If they want to have children, how many children, they sit together and plan that. (PID 4)*

The MHCPs discussed that if their patients did use a contraceptive method, it was typically Nexplanon, birth control pills, condoms, or the pregnancy calendar method. One MHCP said:

*I’ve noticed a lot of them are interested more in the Nexplanon. Some of them ask for the birth control pill. (PID 5)*

Another MHCP stated:

*When it comes to Marshallese, the younger generation they use—they recommend their partners to use protection, condoms and all and that, diaphragms. Also, the ladies or the woman use it over here because they have more education on that subject when they move over here to this state. I will say the older generation they use the—they know their calendar, they use that when to be with their partners, and not when to be with their partners. (PID 4)*

#### 3.3.2. Reproductive Life Planning Influences

MHCPs described similar influences to contraception and reproductive life planning among Marshallese women. Within this theme, three subthemes emerged: (1) Partner; (2) Culture/Religion; and (3) Side Effects.

Partner. The MHCPs described Marshallese partners as being a strong influence on contraception and reproductive life planning. One MHCP said:

*I feel like sometimes they feel as if it’s their men’s decision to make that for them. It’s probably more than five kids, and they’ve been having them C-section, and you’re like, ‘Have you thought about birth control?’ They’re like, ‘Oh, well let me just wait for my partner to come in, and then we’ll talk about it.’ I feel like it’s the men’s decisions, so I don’t think they ever think about birth control for themselves. (PID 1)*

Another MHCP said that they hear their patients say, “Or they will honestly tell me like ‘oh, my guy doesn’t like to use that.’” (PID 1)

The discussion about partners as a strong influence on contraceptive use and reproductive life planning was tethered to the inappropriateness of discussing sensitive topics with mixed genders. For example, one MHCP said: “Also, I feel sometimes that men have more say. They answer for them, so I feel like they think that we are taking away some power” (PID 3). Another MHCP said:

*Every time I try to bring it up with my own patients, and I’m trying to get their spouse into the conversation as we’re discussing their different options, they shy away from that kind of giggle and try to find ways to avoid that. They kind of giggle and find ways to avoid that. I think part of it, again, I don’t think they are comfortable. I don’t think it’s a topic that Marshallese couples discuss at length. I think it’s more an expectation of what the women and the men’s roles are supposed to do. (PID 2)*

Culture/Religion. Similar to the Marshallese mothers, the MHCPs described Marshallese culture and religion as a strong influence on contraception and reproductive life planning. Culture as an influence included both a deep religious belief system that encourages large families alongside a cultural belief system of not discussing contraception and reproductive life planning. For example, one MHCP said: “I think it’s that. We believe in this was our purpose. God gave us the ability to produce, and that’s our purpose” (PID 1). Another MHCP stated:

*I know that every time I talk to somebody and ask them if they are planning for more children or something along that line questions, a lot of the answers that I’ve been hearing or responses is more of ‘Well it’s up to God, you know, we can say we plan but at the end of the day, it’s all in God’s hands.’ (PID 5) *

From the perspective of MHCPs, large family size is an aspect of the Marshallese community that negates conversation about contraception and reproductive life planning. One MHCP said:

*I think culturally there’s a custom, the more we have in the family, the richer you are. It’s always culturally, the goal to have a big family, I guess because of the richness of it, but just the passing down of tradition and custom in the Marshallese custom. I do not believe that there is a particular discussion about family planning. There is the name of this discord among the women. As a male Marshallese, it’s not something that we discuss with the wives or with our sisters and parents, brothers. It seems like it’s something that’s expected, build a big family. It’s almost like a natural thing for the men, the males of the community or society or the family. It’s our responsibility to make sure that whatever that family is, the size is, that all is well and we’re doing our part as a household. (PID 2)*

Some MHCPs described that discussing contraception and reproductive life planning is a highly sensitive subject and can create embarrassment when discussed. One MHCP said: “When it comes to Marshallese and their culture this subject is really sensitive to talk about and discuss” (PID 4).

Another MHCP said:

*Another big challenge I see is it’s really hard, and especially when we get embarrassed, we look around to see if anybody is looking. We might want to get that when we go to the store, because we know that we need it, we are planning, but we’re afraid that somebody might look and say, oh, we have this—these thoughts of people are reading our minds, and rushing to go get it, to go buy it. That’s the big challenge. (PID 4)*

Discussing contraceptives and reproductive life planning was described as difficult, even when Marshallese men are not present. One MHCP said, “From my experience, from what I’ve seen around or encountered with maybe a few or more Marshallese women, it doesn’t seem like it’s an easy open conversation between uh us Marshallese women” (PID 05). However, some MHCPs described seeing a change generationally with comfortability in having open dialogue about contraception and reproductive life planning. One MHCP said:

*I really don’t know the old ways. Like I said, I don’t think family planning is a thing in our culture in terms of the number of people in the family. I think, at least nowadays when people want to delay their family they can seek healthcare. They go to the hospital to see their doctor, or they see if there’s a reproductive clinic. At least in the islands, that’s where they usually go. Either they discuss about family planning, natural methods, or they talk about contraception, whether it’s using condoms or using implants or IUDs or birth control pills. I think nowadays it’s becoming more common, at least more of an individual person or an individual family’s choice of what birth control method they’ll use, that they’re trying to use to delay their pregnancy. (PID 2)*

Another MHCP echoed this sentiment when they stated:

*I mentioned that before the younger generation because they’re to the Americanized ways, and they live in the Western world, and that they can go to any store to get it, the protection for guys, for men to get it if they doing a family planning thing. They also sit together. Like I mentioned before they sit and plan if they are ready to have a family. (PID 4)*

Side Effects. Similar to the Marshallese mothers, the MHCPs described the potential for side effects as a strong influence in contraception use specifically. One MHCP said:

*Is it painful to put in the IUD, or is it painful to put in the implantable device? Then of course other questions usually come up; how does it work? How long does it work for? That doesn’t mean that it’s a permanent thing. If they get one or the other, is it going affect them getting pregnant later? Then basically how effective it is or side-effects, does it cause them to get more bleeding? Does it cause them to have no bleeding or irregular bleeding? Those kind of things are usually the common questions that I get. (PID 1)*

Another MHCP described concerns of side effects from specific contraceptives from their patients:

*Norplant. They have heard all about it back in the islands. I’ve talked to several women, probably more than 10 or close to 20 people. I know that I’ve taken that and they—some of them regret taking that because they heard that it’s for animals. Some of them have side effects not having their period for a longest time after taking it. Some usually have who have regular menstrual period, but after taking it they have abnormal, not normal. (PID 4)*

Implants were also discussed as a form of contraception that their Marshallese patients had concerns regarding the side effects. For example:

*The thing about implants are it stays on for three to five years or longer, some complain that the ones they’ve had which have been over maybe three or five years, it causes side effects to them like, one of them complained about constant head ache, so side effects are what they complain about for the ones that have it already. (PID 5)*

## 4. Discussion

### 4.1. Principal Findings

The purpose of this study is to understand Marshallese mothers’ and MHCP practices and influences with contraception use and reproductive life planning. In this study, many of the Marshallese mothers described not using contraception or reproductive life planning practices despite having a broad understanding of what methods can be used. Much of their knowledge about contraception and reproductive life planning practices was obtained from their MHCPs. 

### 4.2. Results

Numerous influences were discussed in contraception and reproductive life planning decision making. The most dominant influences were partners/family, religion, the potential for side effects from contraceptives, cost, and fear. Similar to previous studies with Native Hawaiian and Pacific Islanders, the dominant influences of partners and religion were embedded in cultural belief systems of not discussing sex or reproductive life planning concepts [21,23]. Spousal communication has been identified in other collectivist cultures as influential in contraception use and reproductive live planning practices [31,32,33]. 

Contraceptive use as part of reproductive life planning practices was also influenced by the potential for negative side effects, especially the fear of infertility. These discussions are likely predicated on lack of health literacy and/or Marshallese customs of not discussing reproductive life planning practices, with the addition of a desire for large families. Fear of contraceptive side effects and infertility is not uncommon and has been previously identified among ethnic and/or minority women [34,35], more specifically with collectivist women who may place more value on large families [31,32,33]. 

An expected influence in contraception and reproductive life planning practices among Marshallese mothers was the lack of insurance and inconsistent means to afford healthcare visits or contraception. This finding is consistent with prior literature which has shown that cost and lack of insurance constrains maternal care and chronic disease management among Marshallese in the US [22,29,36]. This finding is also consistent with prior literature in other populations which has shown reduced contraceptive uses among low income and uninsured women [37,38]. This article adds new insights on the constraints of lack of insurance in contraception and reproductive life planning practices for the Marshallese, as this community has not had access to health insurance outside of pregnancy until December 2020.

Marshallese mothers also described fear as a guiding influence in their contraception use and reproductive life planning practices. Similar to the discussion around the potential for negative side effects from contraceptives, fear was embedded in a lack of contraception health literacy, fear of talking to their healthcare provider, and Marshallese customs of not discussing contraception and reproductive life planning practices. Importantly, fear was ubiquitous in all the influences highlighting a complex challenge for contraception and reproductive life planning education within this community. Although these fears have not been documented in other Pacific Islander cultures, studies with other collectivist cultures such as those in Ethiopia, Kenya, and Pakistan have similarly identified fear of infertility and side effects from contraceptives as highly influential in contraception use and reproductive life planning practices [31,32,33].

Similar to the Marshallese mothers, MHCPs described many of their Marshallese patients as not using contraception or reproductive life planning practices. The MHCPs described this as generational, with younger generations of Marshallese women more open and more informed about contraception and reproductive life planning practices. Previous studies with Native Hawaiian and Pacific Islanders did not identify a generational difference in openness to discussing contraception and reproductive life planning practices. This suggests an opportunity to tailor interventions based on this generational divide among Marshallese communities [22,23].

MHCPs identified similar influences on contraception use and reproductive life planning practices among their Marshallese patients, and these included partners, culture/religion, and side effects. MHCPs described partners as a strong influence on contraception use and reproductive life planning practices and decision making. Similar to the Marshallese mothers, much of the discussion around partner influence included cultural customs of not discussing contraception use or reproductive life planning practices, but they also described partners as the most influential decision maker. The MHCPs expanded on the cultural beliefs around not discussing contraception and reproductive life planning practices and highlighted that in Marshallese culture, it is highly encouraged to have large families. The encouragement of large families was described as a way to pass down the traditions and customs of Marshallese culture. 

Lastly, the MHCPs also identified the potential for contraception side effects as a strong influence on their Marshallese patients. Similar to the mothers, much of the discussion around side effects appears to emerge from a lack of health literacy and misconceptions and/or misinformation about contraception. A global systematic review of MHCPs identified that fear of contraception side effects and low health literacy about contraception was highly influential in contraception and reproductive life planning practices [35].

### 4.3. Clinical Implications

Health interventions not aligned with cultural values and perspectives of the target population are less effective than culturally responsive interventions that account for these factors [39,40]. Culturally-adapted approaches using community-based assets and Marshallese cultural values/practices have been demonstrated to be effective in improving healthcare [41,42] but have not been focused on contraception and reproductive life planning among Marshallese in the US. The results from this study will inform the development of a culturally-adapted contraception and reproductive life planning tool and education program for Marshallese women, couples, and health care providers. 

### 4.4. Research Implications

Additional research is needed in several key areas. Future research should explore contraception and reproductive life planning practices and influences with Marshallese, and MHCPs that work with Marshallese communities, outside of Arkansas. Additionally, future research should consider conducting focus groups rather than individual interviews to foster a robust reciprocal conversation and comfortability. Lastly, future research should examine the variances among Marshallese mothers’ responses based on different age brackets and length of time in the US to explore the effects of generational beliefs and acculturation on practices and influences with contraception use and reproductive life planning. 

### 4.5. Strengths and Limitations

This study’s findings should be evaluated with some limitations. All participants were recruited from Arkansas, and the result may or may not be generalizable to other Pacific Islander communities residing outside of Arkansas. Although the qualitative methods used allow participants to explore the research topic in their own words, participants may have tailored their responses for acceptability. Despite these limitations, this is the first study to document Marshallese mothers’ and MHCPs’ practices and influences with contraception use and reproductive life planning, thus adding substantially to the literature gap. 

## 5. Conclusions

Although the literature on Pacific Islanders’ contraception and reproductive life planning practices and influences is scarce, studies have identified similar practices and influences to those found in this study with the Marshallese community residing in Arkansas [21,22,23]. Similarities included partner influence, lack of health literacy, and cultural customs of not discussing contraception use and reproductive life planning methods [21,22,23]. However, this study adds to the gap in literature by identifying unique challenges among Marshallese, including challenges with cost/health insurance and an overall fear of the process. The results from this study will inform the development of a culturally-adapted contraception and reproductive life planning tool and education program for Marshallese women, couples, and healthcare providers. 

## Figures and Tables

**Table 1:** Marshallese Mothers Demographics.

	Frequency	%
Gender		
Female	15	100
Total	15	100
Ethnicity		
Marshallese	15	100
Total	15	100
Pregnancy Status		
Not pregnant	15	100
Total	15	100
Relationship Status		
Married	3	20.0
Separated	1	6.7
Never married	1	6.7
Unmarried couple	10	66.7
Total	15	100
Education		
Some high school (grades 9–11)	2	13.3
High school graduate (grade 12 or GED)	6	40.0
Some college or technical school (college 1–3 years)	6	40.0
College graduate (4 years or more)	1	6.7
Total	15	100
Age (years)		
Mean	30	---
Standard deviation	7	---
Range	20–42	---

GED = graduate equivalency degree.

**Table 2:** Maternal Healthcare Provider Demographics.

	Frequency
Gender	
Female	3
Male	2
Total	5
Provider Specialty Type	
Clinical Nurse	2
Outreach Specialist	1
Disease Intervention Specialist	1
Family Medical Doctor	1
Total	5
Number of Years Practiced	
Average	7
Minimum	3
Maximum	15

## Data Availability

The deidentified data underlying the results presented in this study may be made available upon request from the corresponding author, Britni L. Ayers, at blayers@uams.edu. The data are not publicly available in accordance with funding requirements and participant privacy.

## Supplementary Materials

The following supporting information can be downloaded at: https://www.mdpi.com/article/10.3390/ijerph20053949/s1, Interview Guide S1: Focus Group/Interview Discussion Guide for Reproductive Life Planning Health Care Professionals.
