# Supplementary material for: Marshallese Mothers’ and Marshallese Maternal Healthcare Providers’ Perspectives on Contraceptive Use and Reproductive Life Planning Practices and Influences
Source: Int J Environ Res Public Health. 2023 Feb 23;20(5):3949. doi: 10.3390/ijerph20053949 (PMC10001766; doi:10.3390/ijerph20053949)
Supplement: Supplementary file 1 [file ijerph-20-03949-s001.zip › ijerph-2118976-supplementary.pdf]

FOCUS GROUP/INTERVIEW DISCUSSION GUIDE FOR REPRODUCTIVE LIFE PLANNING
HEALTH CARE PROFESSIONALS

INTRODUCTION

The purpose of our discussion today is to identify what challenges exist in reproductive life planning for Marshallese women in our community. Our discussion should take about one hour. Please know that your answers will be kept completely private. That means we will not use your name or identify you or link your answers to your identity.

We are interested in your opinion, and there are no right or wrong answers. If you don't understand a question, let me know and I can ask it another way. Some of the topics that we will be discussing may be sensitive. If you are uncomfortable with any of the topics, you do not need to participate in that part of the discussion. I also want to let you know that we will be tape recording this session and taking notes, so that we can be sure to capture all of your comments. This is also why we want you to avoid using your own name or others' names.

PREGNANCY

1. How do Marshallese women talk about planning a family?
   - *Probe:*
     a. *How do Marshallese women generally discuss plans to have children/delay having children?*
2. If a Marshallese woman wants to delay or prevent pregnancy, what does she typically do?

TYPES OF BIRTH CONTROL

3. Can you tell me about the types of birth control methods you prescribe or advise to Marshallese women?
   - *Probe:*
     a. *What challenges/successes have you experienced prescribing or advising these methods?*

MODERATOR: **Now, I'm going to ask you about long-acting reversible contraceptives (LARC), specifically IUDs and birth control implants.**

4. What have Marshallese women heard about IUDs?
   - *Probes:*
     a. *Do you think Marshallese women are generally familiar with or have heard of IUDs?*
     b. *What have you heard from Marshallese patients about IUDs? What questions have Marshallese patients asked you?*
5. What have Marshallese women heard about birth control implants?
   - *Probes:*
     a. *Do you think Marshallese women are generally familiar with or have heard of implants?*
     b. *What have you heard about birth control implants from Marshallese patients? What questions have Marshallese patients asked you?*

6. How comfortable do you think Marshallese women are talking with their health care providers about LARC?
   - *Probe:*
     a. *Why or why not?*

ACCESS/USE OF BIRTH CONTROL

MODERATOR: **Next, I have a few questions about birth control in general, including not only long-acting reversible contraceptives, but also things like condoms, pills, patches, rings, injectables, spermicide, natural family planning, withdrawal, abstinence, men having a vasectomy, women having a tubal ligation, and others.**

7. In your role as a medical professional when thinking about Marshallese patients you may know who are currently using birth control, what types of birth control do they use? Which types of birth control are most common?
   - *Probe:*
     a. *Please describe what you know about their experiences getting birth control.*
        i. *Did they face any difficulties or barriers?*
8. What are some of the things that make it **hard** for Marshallese women in your community to get birth control if they want to prevent pregnancy?
   - *Probes:*
     a. *What is the impact of religion?*
     b. *What is the impact of language barriers?*
     c. *How do fears of side effects play a role?*
     d. *How does limited access to health care and/or contraception affect it?*
     e. *How does cost affect their decision?*
     f. *How do Marshallese women feel discussing it with their partners?*
9. What are some of the things that make it **easy** for Marshallese women in your community to get birth control if they want to prevent pregnancy?
10. How do Marshallese women in your community learn about birth control?
    - *Probe:*
      a. *Who do Marshallese women ask about birth control?*

HEALTHY START PROGRAM

MODERATOR: **We would like to ask you about the Jined ilo Kobo Healthy Start program.**

11. What have you heard about the Jined ilo Kobo Healthy Start Program?
    - *Probe:*
      a. *Where did you hear about it? From another health care provider, on the radio, posters, or other method?*
12. What do women in your community think about the Jined ilo Kobo Healthy Start Program?
    - *Probe:*
      a. *Have you heard good things? Bad things?*
13. Do you know of anyone who has used Jined ilo Kobo Healthy Start Program services? If so, what have you heard from patients about their experiences with Healthy Start?

14. Would you recommend your patients to participate in a program like the Jined ilo Kobo Healthy Start program? Why or why not?

Concluding Question

MODERATOR: **I want to make sure that I captured everything you wanted to say correctly. Is there anything else you would like for me to know about contraceptive access for Marshallese patients in Northwest Arkansas?**

CLOSING: **I want to thank you for participating in this focus group/interview session today. If you are interested in learning more about the Jined ilo Kobo Healthy Start Program, we are happy to provide you with additional information, as well as a contact so that you can learn how to refer patients to the Jined ilo Kobo Healthy Start program.**
